# Adherence to a Mediterranean diet and long-term changes in weight and waist circumference in the EPIC-Italy cohort

**DOI:** 10.1038/s41387-018-0023-3

**Published:** 2018-04-25

**Authors:** Claudia Agnoli, Sabina Sieri, Fulvio Ricceri, Maria Teresa Giraudo, Giovanna Masala, Melania Assedi, Salvatore Panico, Amalia Mattiello, Rosario Tumino, Maria Concetta Giurdanella, Vittorio Krogh

**Affiliations:** 1 Epidemiology and Prevention Unit, Fondazione IRCCS Istituto Nazionale dei Tumori, Milan, Italy; 2 Unit of Epidemiology, Regional Health Service, Grugliasco, Turin, Italy; 3 Department of Mathematics, University of Turin, Turin, Italy; 4 Cancer Risk Factors and Lifestyle Epidemiology Unit, Cancer Research and Prevention Institute (ISPO), Florence, Italy; 5 Department of Clinical and Experimental Medicine, Federico II University of Naples, Naples, Italy; 6 Cancer Registry, Provincial Health Unit (ASP) Ragusa, Ragusa, Italy

## Abstract

Excessive calorie intake and physical inactivity are considered key determinants of the rapid worldwide increase in obesity prevalence, however the relationship between diet and weight gain is complex. We investigated associations between adherence to a Mediterranean diet and long-term changes in weight and waist circumference in volunteers recruited to the Italian section of the prospective European Prospective Investigation into Cancer and Nutrition (EPIC). We investigated 32,119 cohort members who provided anthropometric measures at recruitment and updated information on recall a mean of 12 years later. Adherence to a Mediterranean diet was assessed using the Italian Mediterranean Index (score range 0–11). Associations between index score and weight and waist changes were assessed by multivariate linear regression models. Risks of developing overweight/obesity and abdominal obesity were investigated by multivariate logistic models. Increasing Italian Mediterranean Index score (indicating better adherence) was associated with lower 5-year weight change in volunteers of normal weight at baseline (β −0.12, 95% CI −0.16 to −0.08 for 1 tertile increase in score), but not in those overweight/obese at baseline (P interaction between Index score and BMI 0.0001). High adherence was also associated with reduced risk of becoming overweight/obese (OR 0.91, 95% CI 0.84–0.99 third vs. first tertile); smaller 5-year change in waist circumference (β −0.09, 95% CI −0.14 to −0.03 for 1 tertile increase in score); and lower risk of abdominal obesity (OR 0.91, 95% CI 0.84–0.99 third vs. first tertile). Adherence to a traditional Italian Mediterranean diet may help prevent weight gain and abdominal obesity.

## Introduction

In the three decades or so up to 2013, the global prevalence of overweight and obesity rose by about 28% in adults and 47% in children, from 857 million in 1980 to 2.1 billion in 2013^1^. Data from the National Health and Nutrition Examination Survey indicate that between 1999 and 2008 the prevalence of abdominal obesity in the United States increased by about 6% in men and women^2^. General and abdominal obesity are both associated with increased mortality and incidence of chronic diseases^3–5^.

In strictly logical terms obesity arises when calorie intake remains in excess of calories expended for an extended period. The excess has been estimated as substantial (400 kcal/d) in the United States and is considered due to dietary patterns involving energy overconsumption, as well as increase in sedentary lifestyle^6^. Nevertheless some people are more susceptible to weight gain than others, and the overall pattern of diet is suspected to influence whether or not a person becomes obese^6^.

Analysis of dietary patterns has emerged as a useful approach to examine  relationships between diet and chronic diseases^7^, and also weight change^8^. A 2011 systematic review of randomized controlled trials^9^ found that following a Mediterranean-type dietary pattern could help reduce body weight, but only if associated with restricted energy intake or increased physical activity. However, evidence on the effects of diet and lifestyle on body weight specifically in the Italian population is scant, and only few studies—all on Italians at high risk of overweight/obesity—have been conducted^10–13^.

The aim of the present study was to assess associations between adherence to Mediterranean diet, as assessed by the Italian Mediterranean Index^14^, and subsequent long-term changes in weight and waist circumference, and also risk of developing overweight/obesity, in volunteers recruited to EPIC-Italy.

## Materials and methods

### Study population

EPIC-Italy is the Italian section of the European Prospective Investigation into Cancer and Nutrition (EPIC), a multicenter prospective cohort study investigating the role of metabolic, dietary, lifestyle, and environmental factors in the development of cancer and other chronic diseases. In 1993–1998, 47,749 healthy volunteers were enrolled in EPIC-Italy from five centres: Varese and Turin (Northern Italy), Florence (Central Italy), and Naples and Ragusa (Southern Italy). Details of recruitment and study design are presented elsewhere^15^. The overall EPIC project was approved by the ethics committee of the International Agency for Research on Cancer (IARC), Lyon, France, and in Italy by the ethics committee of the Local Health Authority of Florence. All participants gave informed consent to use clinical data for research.

Follow-up lifestyle and anthropometric information was obtained from EPIC-Italy participants through questionnaires. Mailed questionnaires were used in Turin and Florence; telephone interviews in Ragusa and Naples; and a combination of both was used in Varese. For logistic reasons follow-up started initially (2004) in Florence, and lastly in 2008 in Turin, thereby occurring from 6 to 20 years after recruitment.

A total of 44,503 volunteers were apparently eligible for recall, after excluding those lost to follow-up, who had died or moved out of the area, or—in Varese and Florence—who had been diagnosed with cancer at a previous follow-up. However on attempting re-contact we found that 10,575 additional persons had died, moved away, or refused to participate. Thus, 33,478 persons were contacted and provided update information.

For the present study, we also excluded those who did not complete the baseline food frequency (FFQ) or lifestyle questionnaires (*n* = 320); those with implausible self-reported weight at recall, and those with a ratio of total energy intake (determined from baseline FFQ) to basal metabolic at either extreme of the distribution (cut-offs first and last half-percentiles), leaving 32,262 persons. Finally, we excluded those with missing information on weight change (either because baseline or follow-up weight was missing) or confounder variables (*n* = 143). Thus, 32,119 persons (9662 men and 22,457 women) were included in the present analysis.

### Anthropometric variables at baseline and follow-up

At baseline, anthropometric measurements including weight, height, and waist circumference were measured in light clothes, without shoes, by trained personnel. At follow-up, self-reported measurements were provided including waist circumference (Varese, Turin, and Florence only) and weight (all centres). Changes in waist circumference were analysed in 22,919 volunteers from Varese, Turin, and Florence. Changes in weight were analysed in all 32,119 participants. BMI was weight (kg) divided by height (m) squared. Follow-up BMI employed weight reported at follow-up and height measured at baseline.

Because of the large variation in time between recruitment and follow-up, we assessed 5-year weight change as the difference between weight at follow-up and weight at baseline divided by follow-up time (years) and multiplied by five. We used a similar method to calculate a 5-year change in waist measurement (cm). We also assessed risk of becoming overweight/obese (BMI ≥ 25 kg/m^2^) in those of normal weight (BMI < 25 kg/m^2^) at baseline, and risk of abdominal obesity (waist>102 cm for men and >88 cm for women) in those with waist circumference below these cut-offs at baseline.

### Dietary variables

The frequency of consumption of food items was investigated by validated^16^ semi-quantitative FFQs designed to capture local eating behaviour: one for northern/central Italy (Varese, Turin, and Florence), one for Ragusa, and one for Naples. The questionnaires contained questions on 188, 217, and 140 food items, respectively, and investigated diet over the 12 months preceding administration. Next the food items were linked, using specific software^17^, to Italian Food Tables^18^ in order to obtain estimates of daily energy intake (and 37 macro and micro-nutrients).

Adherence to a Mediterranean diet was assessed using the Italian Mediterranean Index^14^, whose score is calculated from the intake of 11 items: high intake of six typical Mediterranean foods (pasta, typical Mediterranean vegetables (raw tomatoes, non-brassica leafy vegetables, courgettes, aubergines, peppers, onion and garlic), fruit, pulses, olive oil, and fish); low intakes of four “non-Mediterranean” foods (soft drinks, butter, red and processed meat, and potatoes) and moderate consumption of alcohol. For consumption of each typical Mediterranean food in the highest tertile of the consumption distribution, a person received 1 point; consumption in the other two tertiles received zero points. For consumption of non-Mediterranean foods in the lowest tertile of the distribution 1 point was awarded; consumption in the other two tertiles received zero points. Alcohol received 1 point for intake >0 up to 12 g/day; abstainers, and persons who consumed >12 g/day received zero points. Possible scores are in the range 0–11.

### Other covariates

Data on reproductive and medical history, alcohol consumption, smoking, physical activity, education, and other socioeconomic variables were collected at baseline from each participant using a standardized lifestyle questionnaire^19^.

### Statistical analyses

Baseline characteristics, by tertiles of Italian Mediterranean Index, were abridged as means and standard deviations (continuous variables) or frequencies (categorical variables). ANOVA was used to compare means and the chi-square test used to compare proportions.

Multivariate linear regression models were used to assess the relation between the Italian Mediterranean Index score (tertile increase in score) and 5-year weight change. We used a minimally adjusted model, with centre, age, sex, and non-alcoholic intake of energy as covariates; and also a fully adjusted model, in which time from baseline to follow-up (years, continuous), baseline BMI (<25 kg/m^2^, 25 to <30 kg/m^2^, ≥30 kg/m^2^), education (≤8 years, >8 years), menopausal status in women (perimenopausal, premenopausal, and postmenopausal) and physical activity (active, moderately active, moderately inactive, and inactive) were additional covariates. We explored effect modification by sex and baseline BMI by adding, to the model, product terms for Italian Mediterranean Index (continuous, 1 tertile increase) and sex or BMI (categorical), and performing the Wald test. Since *P* for interaction of Italian Mediterranean Index score with sex was not significant, we did not perform separate analyses for men and women.

For persons of normal weight (BMI<25 kg/m^2^) at baseline logistic regression was used to explore the relation of Italian Mediterranean Index score to risk of becoming overweight/obese, estimating odds ratios (ORs) with 95% confidence interval (CI) for each one-point increase in the Index score, and for tertiles of Index score. Trends of linearity of across tertiles were tested by treating each tertile as a continuous variable in the logistic model. Minimally and fully adjusted models were run, using the same covariates as for the linear regression models (except baseline BMI).

To gauge the effect of each component of the Italian Mediterranean Index on risk of becoming overweight/obese, we subtracted each component, one at a time, from the original score (so that score range was reduced to 0–10) and ran the models.

Finally, we used linear and logistic models, as described above, to probe the association of Italian Mediterranean Index score with 5-year change in waist circumference and risk of abdominal obesity. These analyses differed from those on weight change and overweight/obesity in that the fully adjusted models were adjusted for waist circumference at baseline (continuous) instead of baseline BMI; we ran another model that also adjusted for baseline BMI.

All statistical tests were two-sided. Differences were considered significant when *P* < 0.05. The analyses were performed with Stata version 14.2 (College Station, TX, USA).

## Results

After a mean of 11.97 years, mean 5-year weight increase was 0.67 kg, and the mean 5-year waist increase was 3.37 cm. Of the 15,016 volunteers who were normal weight at baseline, 3563 became overweight/obese; of the 19,405 without abdominal obesity at baseline, 5778 became abdominally obese; of the 17,103 volunteers overweight/obese at baseline, 1597 became normal weight at recall.

The baseline characteristics of the participants, according to tertiles of the score of the Italian Mediterranean Index, are shown in Table 1. Age and non-alcoholic energy consumption generally increased with increasing Index score. Persons with greatest Italian Mediterranean Index score were more likely to be female, well-educated, non-smokers, and have higher baseline BMI; women were more likely to be postmenopausal. Persons of the Florence and Naples centres had higher Index score than those at other centres.

**Table 1:** Baseline characteristics of study participants according to tertiles of score of Italian Mediterranean Index

	Tertile I, score 0–3 (*n* = 12,831)	Tertile II, score 4–5 (*n* = 12,134)	Tertile III , score 6–11 (*n* = 7154)
		Mean (SD)^a^	
Age (years)	49.9 (7.7)	50.5 (7.7)	51.0 (7.7)
Non-alcoholic energy intake (Kcal/d)	2154 (591)	2271 (688)	2417 (676)
Baseline to follow-up interval (years)	12.0 (2.4)	12.0 (2.3)	11.9 (2.2)
		*N* (%)^a^	
Males	4231 (33.0)	3593 (29.6)	1838 (25.7)
Centre			
Turin	2571 (20.0)	2367 (19.5)	1319 (18.4)
Varese	3377 (26.3)	2586 (21.3)	1244 (17.4)
Florence	4114 (32.1)	3910 (32.2)	2502 (35.0)
Naples	586 (4.6)	1699 (14.0)	1533 (21.4)
Ragusa	2183 (17.0)	1572 (13.0)	556 (7.8)
Education: ≤8 years	6061 (47.2)	5748 (47.4)	3155 (44.1)
Smoking status			
Current smoker	3495 (27.2)	3082 (25.4)	1686 (23.6)
Ex-smoker	3369 (26.3)	3400 (28.0)	2177 (30.4)
Never smoker	5967 (46.5)	5654 (46.6)	3291 (46.0)
Physical activity			
Inactive	3414 (26.6)	3645 (30.0)	2285 (31.9)
Moderately inactive	5244 (40.9)	4606 (38.0)	2462 (34.4)
Moderately active	2368 (18.4)	2084 (17.2)	1251 (17.5)
Active	1805 (14.1)	1799 (14.8)	1156 (16.2)
BMI			
<25 kg/m^2^	6186 (48.2)	5557 (45.8)	3273 (45.8)
25–29.99 kg/m^2^	5017 (39.1)	4917 (40.5)	2899 (40.5)
≥30 kg/m^2^	1628 (12.7)	1660 (13.7)	982 (13.8)
Postmenopausal women	3764 (43.8)	4005 (46.9)	2564 (49.9)

^a^Tertile values differ significantly (*P* < 0.05) in all cases

Table 2 shows the association between Italian Mediterranean Index score and 5-year weight change overall, and by baseline BMI category. Score was not significantly associated with overall weight change. However, when we stratified according to baseline BMI, in those with baseline BMI < 25 kg/m^2^, a negative change in weight was significantly associated with tertile increases in Index score (β −0.11, 95% CI −0.15 to −0.06, fully adjusted model). The Index score and baseline BMI were found to interact significantly (*P* < 0.0001, fully adjusted model, not shown in tables).

**Table 2:** Association between Italian Mediterranean Index score (continuous variable, per tertile increase) and mean 5-year weight change

	β^a^ (95% CI)	β^b^ (95% CI)
Overall	−0.04 (−0.07, 0.00)	−0.03 (−0.06, 0.01)
Baseline BMI category		
<25 kg/m^2^	−0.12 (−0.16, −0.08)	−0.11 (−0.15, −0.06)
25–29.99 kg/m^2^	0.03 (−0.02, 0.09)	0.04 (−0.01, 0.10)
≥30 kg/m^2^	0.06 (−0.08, 0.20)	0.06 (−0.08, 0.20)

^a^Adjusted for centre, age, sex, and non-alcoholic energy intake

^b^Further adjusted for time from baseline to follow-up, BMI at baseline, education, physical activity, and menopausal status (in women)

Table 3 shows ORs for becoming overweight/obese in relation to complete Index score and after removing from the score one dietary component at a time. High index score was associated with reduced risk of overweight/obesity in models in which score was categorical (tertiles) (OR 0.89, 95% CI 0.80–0.99, *P* trend 0.030) and continuous (OR 0.98, 95% CI 0.95–1.00). However among volunteers overweight/obese at baseline, high Index score was not associated with normalization of BMI, either in the categorical (OR 0.98, 95% CI 0.85–1.13 for the third vs. the first tertile, *P* trend 0.693, fully adjusted model, not shown in tables) or the continuous model (OR 0.99, 95%CI 0.96–1.02, fully adjusted model, not shown in tables).

**Table 3:** Odds ratios (OR) of becoming overweight/obese in relation to score for Italian Mediterranean Index and its components

	*N Cases/controls*	OR^a^ (95% CI)	OR^b^ (95% CI)
Italian Mediterranean Index			
Tertile I (0–3)	1528/4658	1	1
Tertile II (4–5)	1311/4246	0.94 (0.86–1.02)	0.95 (0.87–1.03)
Tertile III (6–11)	724/ 2549	0.88 (0.79–0.97)	0.89 (0.80–0.99)
* P* trend		0.014	0.030
Continuous		0.97 (0.95–0.99)	0.98 (0.95–1.00)
Italian Mediterranean Index minus pasta			
Tertile I (0–3)	1789/5500	1	1
Tertile II (4)	699/2273	0.95 (0.86–1.05)	0.95 (0.86–1.05)
Tertile III (5–10)	1075/3680	0.92 (0.84–1.01)	0.93 (0.85–1.02)
* P* trend		0.061	0.122
Continuous		0.97 (0.95–1.00)	0.98 (0.96–1.00)
Italian Mediterranean Index minus Mediterranean vegetables			
Tertile I (0–3)	1717/5244	1	1
Tertile II (4)	840/2609	0.99 (0.90–1.09)	0.99 (0.90–1.09)
Tertile III (5–10)	1006/3600	0.86 (0.79–0.94)	0.87 (0.79–0.95)
* P* trend		0.002	0.005
Continuous		0.96 (0.94–0.98)	0.96 (0.94–0.98)
Italian Mediterranean Index minus fruit			
Tertile I (0–3)	1756/5730	1	1
Tertile II (4)	742/2387	0.96 (0.87–1.06)	0.96 (0.87–1.06)
Tertile III (5–10)	1065/3696	0.89 (0.82–0.98)	0.90 (0.83–0.99)
* P* trend		0.015	0.032
Continuous		0.97 (0.95–0.99)	0.97 (0.95–0.99)
Italian Mediterranean Index minus pulses			
Tertile I (0–3)	1773/5412	1	1
Tertile II (4)	747/2417	0.95 (0.86–1.05)	0.95 (0.86–1.05)
Tertile III (5–10)	1043/3624	0.90 (0.82–0.99)	0.92 (0.83–1.00)
* P* trend		0.022	0.053
Continuous		0.97 (0.95–0.99)	0.97 (0.95–0.99)
Italian Mediterranean Index minus olive oil			
Tertile I (0–3)	1755/5396	1	1
Tertile II (4)	755/2505	0.94 (0.85–1.04)	0.95 (0.86–1.05)
Tertile III (5–10)	1053/3552	0.91 (0.83–1.00)	0.92 (0.84–1.01)
* P* trend		0.040	0.063
Continuous		0.97 (0.95–0.99)	0.97 (0.95–1.00)
Italian Mediterranean Index minus fish			
Tertile I (0–3)	1767/5328	1	1
Tertile II (4)	753/2422	0.94 (0.85–1.03)	0.94 (0.85–1.04)
Tertile III (5–10)	1043/3703	0.87 (0.80–0.95)	0.88 (0.80–0.96)
* P* trend		0.003	0.005
Continuous		0.96 (0.94–0.98)	0.96 (0.94–0.98)
Italian Mediterranean Index minus potatoes			
Tertile I (0–3)	1832/5585	1	1
Tertile II (4)	646/2203	0.89 (0.80–0.99)	0.89 (0.80–0.99)
Tertile III (5–10)	1085/3665	0.90 (0.82–0.99)	0.91 (0.83–1.00)
* P* trend		0.020	0.037
Continuous		0.97 (0.95–0.99)	0.98 (0.95–1.00)
Italian Mediterranean Index minus butter			
Tertile I (0–3)	1792/5490	1	1
Tertile II (4)	741/2349	0.97 (0.88–1.08)	0.98 (0.89–1.08)
Tertile III (5–10)	1030/3614	0.88 (0.81–0.97)	0.90 (0.82–0.98)
* P* trend		0.011	0.024
Continuous		0.96 (0.94–0.98)	0.97 (0.94–0.99)
Italian Mediterranean Index minus red meat			
Tertile I (0–3)	1816/5600	1	1
Tertile II (4)	688/2285	0.93 (0.84–1.03)	0.93 (0.84–1.03)
Tertile III (5–10)	1059/3568	0.93 (0.85–1.02)	0.94 (0.86–1.03)
* P* trend		0.112	0.154
Continuous		0.99 (0.96–1.01)	0.99 (0.97–1.01)
Italian Mediterranean Index minus soft drinks			
Tertile I (0–3)	1930/6061	1	1
Tertile II (4)	704/2213	0.98 (0.88–1.08)	0.98 (0.89–1.09)
Tertile III (5–10)	929/3179	0.90 (0.82–0.99)	0.92 (0.83–1.01)
* P* trend		0.035	0.083
Continuous		0.97 (0.95–0.99)	0.98 (0.95–1.00)
Italian Mediterranean Index minus alcohol			
Tertile I (0–3)	1915/6045	1	1
Tertile II (4)	725/2194	1.03 (0.93–1.14)	1.03 (0.94–1.14)
Tertile III (5–10)	923/3214	0.91 (0.83–1.00)	0.92 (0.84–1.01)
* P* trend		0.086	0.136
Continuous		0.97 (0.95–0.99)	0.97 (0.95–1.00)

^a^Adjusted for centre, age, sex, and non-alcoholic energy intake

^b^Further adjusted for distance between baseline and recall, education, physical activity, and menopausal status (women)

When individual components of the Index score were excluded, associations changed only minimally: when pasta, pulses, olive oil, soft drinks, and alcohol were excluded, Index score remained significant in the continuous models, but was no longer significantly associated with overweight/obesity in the categorical models. When low red meat intake was excluded, the association between Index score and risk of overweight or obesity was no longer significant in either the categorical or continuous models.

Table 4 displays the association between Italian Mediterranean Index score and subsequent 5-year change in waist circumference. Overall, and for persons with baseline BMI<25 kg/m^2^, each tertile increase in Index score was significantly associated with a negative change in waist circumference (*P* 0.0003 for interaction between Italian Mediterranean Index score and baseline BMI, fully adjusted model, not shown in tables).

**Table 4:** Association between score Italian Mediterranean Index score considered as a continuous variable, per tertile increase) and mean 5-year change in waist circumference

	β^a^ (95% CI)	β^b^ (95% CI)	β^c^ (95% CI)
Overall	−0.09 (−0.14, −0.03)	−0.09 (−0.14, −0.03)	−0.10 (−0.16, −0.05)
Baseline BMI category			
<25 kg/m^2^	−0.20 (−0.27, −0.12)	−0.20 (−0.27, −0.13)	
25–29.99 kg/m^2^	0.00 (−0.09, 0.09)	0.02 (−0.11, 0.07)	
≥30 kg/m^2^	0.12 (−0.09, 0.34)	0.06 (−0.15, 0.27)	

^a^Adjusted for centre, age, sex, and non-alcoholic energy intake

^b^Further adjusted for distance between baseline and recall, waist circumference at baseline, education, physical activity, and menopausal status (women)

^c^Further adjusted for BMI at baseline

Table 5 shows ORs for developing abdominal obesity according to complete Index score and after subtracting each component at a time. High complete Index score was associated with reduced risk of abdominal obesity in both the categorical (OR 0.91, 95% CI 0.84–0.99, *P* trend 0.022, fully adjusted model) and continuous (OR 0.98, 95% CI 0.96–1.00, fully adjusted model) models. The risk decreased further when baseline BMI was added to the model. When pasta, red meat, and soft drinks were excluded, the association was no longer significant (fully adjusted model) or weaker (fully adjusted plus baseline BMI).

**Table 5:** Odds ratios (OR) of developing abdominal obesity in relation to score for Italian Mediterranean Index and its components

	*N* Cases/controls	OR^a^ (95% CI)	OR^b^ (95% CI)	OR^c^ (95% CI)
Italian Mediterranean Index				
Tertile I (0–3)	2488/5658	1	1	1
Tertile II (4–5)	2102/5017	0.93 (0.87–1.00)	0.93 (0.87–1.00)	0.90 (0.83–0.97)
Tertile III (6–11)	1188/2952	0.89 (0.82–0.97)	0.91 (0.84–0.99)	0.86 (0.78–0.94)
* P* trend		0.004	0.022	0.001
Continuous		0.97 (0.96–0.99)	0.98 (0.96–1.00)	0.96 (0.95–0.98)
Italian Mediterranean Index without pasta				
Tertile I (0–3)	2851/6770	1	1	1
Tertile II (4)	1145/2663	0.98 (0.90–1.07)	0.98 (0.90–1.07)	0.96 (0.88–1.05)
Tertile III (5–10)	1782/4194	0.95 (0.89–1.02)	0.97 (0.91–1.05)	0.91 (0.84–0.99)
* P* trend		0.187	0.501	0.025
Continuous		0.98 (0.96–1.00)	0.98 (0.97–1.00)	0.97 (0.95–0.98)
Italian Mediterranean Index without Mediterranean vegetables				
Tertile I (0–3)	2827/6419	1	1	1
Tertile II (4)	1328/3042	0.97 (0.89–1.05)	0.97 (0.90–1.05)	0.97 (0.89–1.06)
Tertile III (5–10)	1623/4166	0.86 (0.80–0.92)	0.87 (0.81–0.94)	0.84 (0.77–0.91)
* P* trend		<0.0001	<0.0001	<0.0001
Continuous		0.96 (0.94–0.98)	0.96 (0.94–0.98)	0.95 (0.93–0.97)
Italian Mediterranean Index without fruit				
Tertile I (0–3)	2811/6364	1	1	1
Tertile II (4)	1205/2921	0.92 (0.85–0.99)	0.92 (0.85–1.00)	0.87 (0.80–0.96)
Tertile III (5–10)	1762/4342	0.90 (0.84–0.97)	0.92 (0.86–0.99)	0.87 (0.81–0.95)
* P* trend		0.004	0.019	0.001
Continuous		0.97 (0.95–0.99)	0.97 (0.96–0.99)	0.96 (0.94–0.98)
Italian Mediterranean Index without pulses				
Tertile I (0–3)	2853/6470	1	1	1
Tertile II (4)	1141/2839	0.89 (0.82–0.97)	0.90 (0.83–0.98)	0.88 (0.80–0.96)
Tertile III (5–10)	1784/4318	0.91 (0.84–0.97)	0.93 (0.86–1.00)	0.88 (0.81–0.95)
* P* trend		0.004	0.029	0.001
Continuous		0.97 (0.96–0.99)	0.98 (0.96–1.00)	0.97 (0.95–0.98)
Italian Mediterranean Index without olive oil				
Tertile I (0–3)	2842/6595	1	1	1
Tertile II (4)	1284/3024	0.96 (0.88–1.03)	0.96 (0.89–1.04)	0.95 (0.87–1.03)
Tertile III (5–10)	1652/4008	0.92 (0.85–0.99)	0.94 (0.87–1.01)	0.89 (0.82–0.96)
* P* trend		0.020	0.076	0.004
Continuous		0.97 (0.95–0.99)	0.98 (0.96–1.00)	0.96 (0.94–0.98)
Italian Mediterranean Index without fish				
Tertile I (0–3)	2849/6420	1	1	1
Tertile II (4)	1157/2856	0.89 (0.82–0.97)	0.89 (0.82–0.97)	0.87 (0.79–0.95)
Tertile III (5–10)	1772/4351	0.88 (0.82–0.95)	0.90 (0.83–0.96)	0.86 (0.79–0.93)
* P* trend		<0.0001	0.002	<0.0001
Continuous		0.96 (0.94–0.98)	0.97 (0.95–0.98)	0.96 (0.94–0.97)
Italian Mediterranean Index without potatoes				
Tertile I (0–3)	2989/6817	1	1	1
Tertile II (4)	1051/2568	0.92 (0.85–1.00)	0.92 (0.85–1.00)	0.90 (0.82–0.99)
Tertile III (5–10)	1738/4242	0.92 (0.85–0.99)	0.94 (0.87–1.01)	0.88 (0.81–0.96)
* P* trend		0.016	0.057	0.002
Continuous		0.97 (0.95–0.99)	0.98 (0.96–0.99)	0.96 (0.94–0.98)
Italian Mediterranean Index without butter				
Tertile I (0–3)	2921/6708	1	1	1
Tertile II (4)	1153/2766	0.93 (0.86–1.01)	0.93 (0.86–1.01)	0.91 (0.83–1.00)
Tertile III (5–10)	1704/4153	0.92 (0.85–0.99)	0.94 (0.87–1.01)	0.90 (0.82–0.97)
* P* trend		0.022	0.082	0.006
Continuous		0.97 (0.95–0.99)	0.98 (0.96–1.00)	0.96 (0.94–0.98)
Italian Mediterranean Index without red meat				
Tertile I (0–3)	2882/6654	1	1	1
Tertile II (4)	1121/2732	0.94 (0.86–1.02)	0.94 (0.86–1.02)	0.89 (0.81–0.98)
Tertile III (5–10)	1775/4241	0.96 (0.89–1.03)	0.97 (0.90–1.05)	0.90 (0.83–0.98)
* P* trend		0.212	0.351	0.009
Continuous		0.99 (0.96–1.01)	1.00 (0.98–1.02)	0.98 (0.96–1.00)
Italian Mediterranean Index without soft drinks				
Tertile I (0–3)	3233/7452	1	1	1
Tertile II (4)	1083/2591	0.95 (0.88–1.03)	0.96 (0.88–1.04)	0.91 (0.83–1.00)
Tertile III (5–10)	1462/3584	0.94 (0.87–1.01)	0.96 (0.89–1.04)	0.90 (0.83–0.98)
* P* trend		0.066	0.270	0.008
Continuous		0.98 (0.96–1.00)	0.99 (0.97–1.00)	0.97 (0.95–0.99)
Italian Mediterranean Index without alcohol				
Tertile I (0–3)	3138/7224	1	1	1
Tertile II (4)	1114/2599	0.97 (0.89–1.05)	0.98 (0.90–1.06)	0.94 (0.85–1.03)
Tertile III (5–10)	1526/3804	0.91 (0.85–0.98)	0.93 (0.86–1.00)	0.89 (0.82–0.97)
* P* trend		0.017	0.059	0.005
Continuous		0.97 (0.95–0.99)	0.98 (0.96–1.00)	0.96 (0.95–0.98)

^a^Adjusted for centre, age, sex, and non-alcoholic energy intake

^b^Further adjusted for distance between baseline and recall, waist education, physical activity, and menopausal status (women only)

^c^Further adjusted for BMI at baseline

## Discussion

We have found that prior adherence to a typical Italian Mediterranean diet, measured with the Italian Mediterranean Index, was associated with a lowered risk of weight gain (in people of normal weight at baseline) and a lowered risk of becoming overweight/obese 6–20 years later. High score on the Italian Mediterranean Index was also associated with lower gain in waist girth and lower risk of abdominal obesity. These associations were marginally influenced by specific components of the Mediterranean diet. Thus, when low red and processed meat consumption was removed as a component of the Index, the resulting diet was no longer “protective” against either overweight/obesity or abdominal obesity. Furthermore when high pasta intake, and low soft drink intake were removed, the resulting diet was no longer protective against abdominal obesity.

These findings are consistent with results of a prospective study on the Spanish EPIC cohort^20^, which assessed adherence to a Mediterranean diet using the Mediterranean Diet Score (MDS)^21^, and found that high MDS was associated with reduced incidence of obesity, but not overweight, over 3 years of follow-up. Furthermore, in the entire European EPIC cohort^22^ a high relative Mediterranean Diet Score (rMED)^23^—indicating good observance of a Mediterranean diet—was associated with reduced weight gain and decreased risk of becoming overweight/obese in persons of normal weight at baseline. Of the components contributing to the rMED score, low consumption of “meat and meat products” had the greatest influence: when removed as a component of the score, the association between high rMED score and low weight gain was no longer significant.

In a study that compared cases (those with greatest weight gain during follow-up) and a sample of non-cases in EPIC cohorts of five countries (Denmark, Germany, Italy, Netherlands, and the UK), higher rMED score was significantly inversely associated with case status (overweight/obesity) but not with change in weight^24^.

Other European cohort studies have also reported inverse associations between adherence to Mediterranean diet and weight gain. A study on the Spanish SUN cohort found that after a mean follow-up of 5.7 (SD 2.2) years, participants with highest adherence—assessed by four instruments (MDS, Mediterranean Adequacy Index, Mediterranean Diet Quality Index, and Mediterranean Dietary Pattern)—had lowest yearly weight gain^25^. In the French SU.VI.MAX cohort, better adherence as measured by the MDS and rMED (but not the Mediterranean-Style Dietary Pattern Score (MSDPS)) was associated with lower 13-year weight change overall and lower risk of obesity in men^26^. By contrast, no association between adherence to the MDS and weight change was found in a Swedish cohort of young women followed for 12 years^27^ or between adherence to a score based on the rationale of the Mediterranean dietary pyramid^28^, or the incidence of obesity in the ATTICA study^29^.

As regards non-European cohorts, Fung et al.^30^ found that increased adherence (over 4 years) to the Alternate Mediterranean Diet was inversely associated with weight gain (same period) in US men and women enrolled in the Nurses’ Health Study, Nurses’ Health Study II, and Health Professionals Follow-Up Study; the association was stronger in younger women and overweight individuals^30^. Population based studies conducted in China^31^ and Iran^32^ found no associations between MDS and weight gain or BMI change, respectively.

Data on waist change and a Mediterranean diet are more limited. The case-cohort study on EPIC participants in five countries found that higher rMED score was inversely associated with increase in waist circumference^24^. A population-based survey in Girona, Spain found that adherence to the validated REGICOR-Mediterranean diet score (assessed in two ways) was inversely associated with increase in waist girth, but not with the incidence of abdominal obesity over 10 years^33^. In the SUN cohort, participants with higher baseline MDS had lower waist circumference after 6 years of follow-up^34^. In the SU.VI.MAX study greater adherence to the MDS and a modified MDS, but not to the MSDPS, was negatively associated with waist circumference after 6 years of follow-up^35^. By contrast the Swedish Women’s Lifestyle and Health cohort study found no association of adherence to MDS with change in waist circumference^27^. It appears that only two prospective studies on Mediterranean diet and waist circumference have been conducted outside Europe: a study on the Framingham Heart Study Offspring Cohort found that US men and women with higher MSDPS had significantly lower waist circumference at follow-up^36^, while the Tehran Lipid and Glucose Study found no association between MDS and waist circumference^32^.

The relation of Mediterranean diet to anthropometric measures has also been investigated in intervention studies. A meta-analysis of 16 randomized controlled trials involving over 3000 persons concluded that a Mediterranean diet could help lower body weight, particularly when diet is energy-restricted and associated with physical activity^9^. A meta-analysis of 11 trials that found that, compared to a control diet, a Mediterranean diet had a beneficial effect on waist circumference^37^.

However results from intervention studies are not directly comparable with those of prospective observational studies, such as the present one, since intervention studies target obese or otherwise highly selected people, and use a specific diet together with strict compliance criteria. By contrast, our study involved a cohort of healthy volunteers in whom following a Mediterranean diet was inferred from the distribution of Italian Mediterranean Index scores in the cohort.

It is not surprising that the inverse association between high Italian Mediterranean Index score and weight increase was present, in our study, only in persons of normal weight at baseline, and was not evident in those who were overweight/obese at baseline. Likely reasons for this are underreporting of both baseline diet and weight at follow-up mainly by overweight/obese participants. In the EPIC-PANACEA study, as our study, the relationship between eating a Mediterranean diet and low weight gain was stronger in low BMI persons but was still significant in overweight persons^22^. In marked contrast, a study on three US cohorts found stronger associations of weight loss to adherence to the diet in overweight/obese than normal weight participants, but this was because the study analysed the effect of change in adherence (not of baseline adherence) to the Mediterranean diet on weight change^30^.

Several mechanisms may explain why a Mediterranean diet protects against weight gain and increasing abdominal girth. First a Mediterranean diet contains a high proportion of foods with low energy-density (water-rich foods, e.g. fruit and vegetables), that induce satiety and hence tend to lower intake of energy. Reducing the energy density of the diet by consuming more fruit and vegetables has been associated with weight loss even in ad libitum diets (reviewed in^38^). Second both low energy-density foods and pulses are rich in fibre, which also contributes to satiety^39^ since fibre-rich foods require more mastication^40^; and increasing the fibre content of a low-fat meal has been reported (in women) to increase cholecystokinin release which appears to mediate the feeling of satiety^41^. Furthermore, short-chain fatty acid production by intestinal bacteria is enhanced by high fibre intake. These fatty acids may have various beneficial effects including activation of hepatic AMP-activated protein kinase. This enzyme regulates metabolic homeostasis and its activation may have favourable effects on abdominal obesity^42^. Finally the Italian Mediterranean diet contains high proportions of pasta and pulses, which are good sources of low glycaemic index carbohydrates^43^. Consuming meals with a lower glycaemic index lowers insulin secretion (an anabolic hormone)^44^, which may contribute to reduced weight gain.

Despite some concerns about the relatively high fat content of a typical Italian Mediterranean diet^45^, most of this fat comes from olive oil and, to a lesser extent, fish. Compared to the saturated fats found in animal foods such as butter and red and processed meat, unsaturated fatty acids promote energy expenditure, diet-induced thermogenesis and fat oxidation^46^, which would explain why a diet that contains a high proportion of unsaturated fatty acids is less prone to cause weight gain. In fact there is evidence that replacing saturated fats with monounsaturated fats leads to weight loss and lower fat mass^47^.

The low red meat content of the Italian Mediterranean diet could also contribute to its beneficial effects on weight change. Data from the EPIC-PANACEA cohort indicate that high meat intake is directly associated with weight gain^48^.

Finally, a Mediterranean diet may reduce gain in waist circumference via an ability to reverse the negative effects due to the 12Ala allele of the PPARgamma gene. The PPARgamma gene product is involved in adipocyte differentiation, lipid storage, and insulin sensitization. The PREDIMED study found that, in 774 persons at high cardiovascular risk, presence of the 12Ala allele was associated with significant gain in waist circumference in the control group, but not in those following Mediterranean-type diets^49^.

Our study has several strengths, including prospective design, use of validated dietary questionnaires to capture dietary intake over the preceding year, and availability of information for several non-dietary variables, making it possible to control for their supposed confounding effects.

However, our study also has several limitations. First, we used self-reported weight at follow-up and there is evidence that self-reporters tend to underestimate their true weight^50^, especially if they are overweight/obese^51^, which may have attenuated associations: in fact, mean 5-year weight increase was low, even though most persons were sedentary (inactive or moderately inactive). Nevertheless, in the EPIC-PANACEA study, after “correction equations” were applied to self-reported weight, the association of high adherence to a Mediterranean dietary pattern with negative 5-year weight change remained^22^. Another limitation is that dietary patterns were inferred from single administration of the FFQ, so that changes in diet over the 6–20 years since FFQ administration were not measured. However dietary pattern assessments are more reliable than assessments of individual foods/nutrients, as indicators of long-term diet^52^. Finally, we cannot exclude residual confounding by factors that we were unable to estimate or estimated sub-optimally in our questionnaires.

To conclude, we have found that following a Mediterranean diet—as measured by an instrument designed to capture key aspects of the traditional Italian Mediterranean diet—is associated with reduced weight gain and reduced increase in waist circumference. Intervention studies are needed to confirm the protective effects of this dietary pattern against weight gain.

## Data availability

The raw data for the present study are deposited with the corresponding author, however their availability is restricted: the ethical committees do not allow open/public sharing of data pertaining to individuals. Aggregated data are available to researchers upon request.
